# Maternal proteomic profiling reveals alterations in lipid metabolism in late-onset fetal growth restriction

**DOI:** 10.1038/s41598-020-78207-3

**Published:** 2020-12-03

**Authors:** Cristina Paules, Lina Youssef, Jezid Miranda, Francesca Crovetto, Josep Maria Estanyol, Guerau Fernandez, Fatima Crispi, Eduard Gratacós

**Affiliations:** 1 Department of Maternal-Fetal Medicine (ICGON), BCNatal|Fetal Medicine Research Center (Hospital Clínic and Hospital Sant Joan de Déu), Institut D’Investigacions Biomèdiques August Pi i Sunyer (IDIBAPS), University of Barcelona, Sabino de Arana 1, 08028 Barcelona, Spain; 2 Instituto de Investigación Sanitaria Aragón (IISAragon), Zaragoza, Spain; 3 Centres Cientifics i Tecnològics (CCiTUB), University of Barcelona, Barcelona, Spain; 4 Bioinformatics Unit, Genetics and Molecular Medicine Service, Hospital Sant Joan de Déu, Esplugues de Llobregat, Spain; 5 Centre for Biomedical Research on Rare Diseases (CIBER-ER), Madrid, Spain

**Keywords:** Medical research, Pathogenesis

## Abstract

Fetal growth restriction defined as the failure to achieve the fetal genetic growth potential is a major cause of perinatal morbidity and mortality. The role of maternal adaptations to placental insufficiency in this disorder is still not fully understood. We aimed to investigate the biological processes and protein–protein interactions involved in late-onset fetal growth restriction in particular. We applied 2D nano LC–MS/MS proteomics analysis on maternal blood samples collected at the time of delivery from 5 singleton pregnancies with late-onset fetal growth restriction and 5 uncomplicated pregnancies. Data were analyzed using R package “limma” and Ingenuity Pathway Analysis. 25 proteins showed significant changes in their relative abundance in late-onset fetal growth restriction (p value < 0.05). Direct protein–protein interactions network demonstrated that Neurogenic locus notch homolog protein 1 (NOTCH1) was the most significant putative upstream regulator of the observed profile. Gene ontology analysis of these proteins revealed the involvement of 14 canonical pathways. The most significant biological processes were efflux of cholesterol, efflux of phospholipids, adhesion of blood cells, fatty acid metabolism and dyslipidemia. Future studies are warranted to validate the potential role of the detected altered proteins as potential therapeutic targets in the late-onset form of fetal growth restriction.

## Introduction

Fetal growth restriction (FGR) is one of the established pregnancy complications that involves fetal distress due to the failure in achieving the fetal genetic growth potential. FGR, usually defined as a birthweight less than 10th centile, affects 7–10% of pregnancies and implicates an increased risk of perinatal morbidity and mortality^1,2^. This condition is also associated with cardiovascular, metabolic and neurodevelopmental changes in the offspring that persist into adulthood^3–5^. Late-onset form of FGR, usually diagnosed after 32 weeks of gestation and delivered at term^6^, is the most common clinical presentation of this condition encompassing more than 90% of FGR cases^7^ and constituting a major contributing factor to adverse perinatal outcome^8^. Placental dysfunction is the main culprit in FGR causing an impairment in the transfer of nutrients and oxygen from the mother to the developing fetus^9^. Maternal adaptations to placental insufficiency may also play a role in the pathophysiology of FGR^10^. Thus, exploring biological pathways in the maternal blood in pregnancies complicated by late-onset FGR may help in identifying the involved etiological mechanisms and detecting potential therapeutic targets for this disorder with the aim of preventing its short and long-term consequences.

Proteomic profiling exemplify the study of the global set of proteins in a particular biosample^11^. Its application in pregnancy-related disorders has been implemented to improve the understanding of their pathophysiology^12^. However, a handful number of previous studies have exploreed the maternal proteomic fingerprint of FGR^13–15^. Moreover, none of them investigated separately late-onset FGR, indeed the studied population in the literature was principally formed by early-onset FGR cases since it’s the most severe phenotype. This approach carries a certain bias given that late-onset FGR might have a different pathogenesis than its early-onset counterpart with similar long-term consecuences^7^.

Our objective in this study was to focus on late-onset FGR and to analyze the maternal blood proteome in pregnancies complicated by this disorder compared to healthy pregnancies in order to determine the biological processes and protein–protein interactions involved in late-onset FGR.

## Results

### Clinical characteristics of the study population

Maternal baseline characteristics were similar between the study groups with the exception of lower maternal age in FGR cases compared to controls, as shown in Table 1. None of the patients included in our study suffered from chronic hypertension or pregestational diabetes. In addition, all of the pregnancies were conceived naturally without the use of assisted reproductive technologies. No differences were observed between the cases and the controls regarding feto-placental Doppler parameters. All the patients included in this study had normal feto-placental Doppler with the exception of one FGR case that presented abnormal cerebroplacental ratio.

**Table 1:** Baseline characteristics and perinatal outcomes of the study populations.

	Uncomplicated pregnancies (n = 5)	Fetal growth restriction (n = 5)	p value
**Baseline characteristics**			
Age (years)	31 (28 to 31)	26.5 (24.5 to 28)	0.05
Caucasian (%)	74	100	0.24
Nulliparity (%)	40	40	1
Smoking (%)	0	20	0.29
**Feto-placental Doppler before delivery**			
Uterine arteries mean pulsatility index (z score)	− 0.27 (− 2.19 to 1.25)	0.14 (− 0.35 to 2.47)	0.65
Umbilical artery pulsatility index (z score)	0.16 (− 0.4 to 0.88)	0.66 (− 0.14 to 1.35)	0.65
Middle cerebral artery pulsatility index (z score)	− 0.33 (− 0.4 to 0.51)	0.25 (− 0.47 to 0.46)	1
Cerebroplacental ratio (z score)	− 0.02 (− 1.32 to 0.49)	− 1.05 (− 1.49 to − 1)	0.46
**Perinatal outcomes**			
Gestational age at delivery (weeks)	39 (38 to 39)	37 (37 to 38)	0.24
Birthweight (gr)	3276 (3030 to 3670)	1980 (1980 to 2420)	0.01
Birthweight centile	42 (41 to 55)	0 (0 to 2)	0.01
Male gender (%)	40	60	0.53
Cesarean section (%)	20	0	0.29
APGAR score 5 min < 7	0 (0)	0 (0)	1
Umbilical cord artery pH	7.28 (7.25 to 7.35)	7.11 (7.11 to 7.12)	0.01

Data are shown as median (interquartile range) or percentages as appropriate. p value was calculated by Mann Whitney U test and Fisher exact test for continuous and categorical variables respectively.

In terms of perinatal outcomes, all the included gestations were delivered at term (> 37 weeks) with no difference between cases and controls (p = 0.24). In accordance with the study design, FGR newborns had significantly lower birthweights (p = 0.01) with birthweight centiles < 3rd centile in all the cases (p = 0.01) compared to controls. No cases of perinatal mortality were observed in the study population.

### Proteomics results

**Figure 1:**
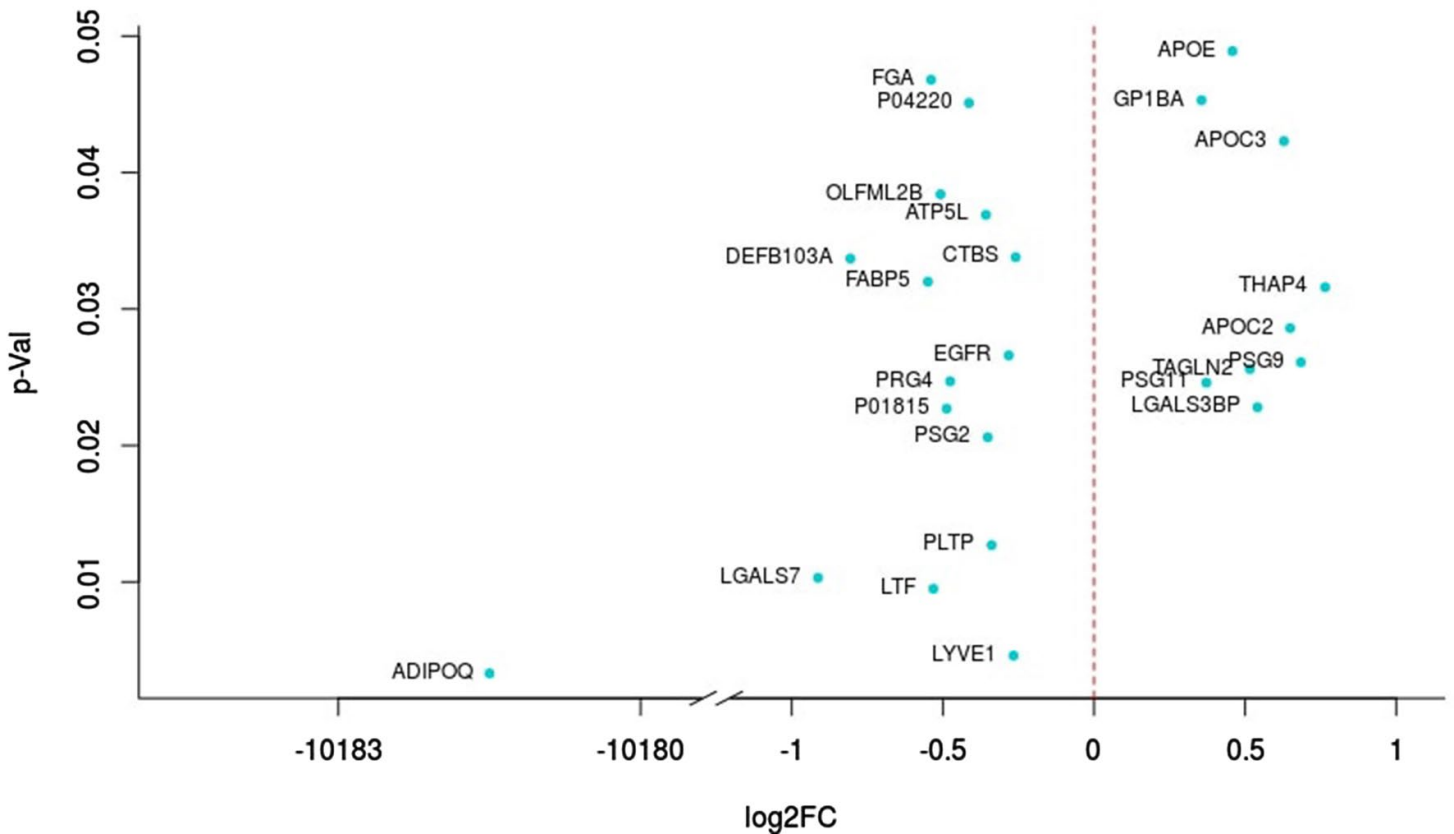
Differentially expressed proteins in late-onset fetal growth restriction. *ADIPOQ* adiponectin, *APOC2* apolipoprotein C-II, *APOC3* apolipoprotein C-III, *APOE* apolipoprotein E, *ATP5L* ATP synthase subunit g mitochondrial, *CTBS* Di-N-acetylchitobiase, *DEFB103A* beta-defensin 103, *EGFR* epidermal growth factor receptor, *FABP5* fatty acid-binding protein 5, *FGA* fibrinogen alpha chain, *GP1BA* platelet glyprotein Ib alpha chain, *LGALS3BP* galectin-3-binding protein, *LGALS7* galectin-7, *LTF* lactotransferrin precursor, *LYVE1* lymphatic vessel endothelial hyaluronan receptor 1, *OLFML2B* olfactomedin-like-protein 2B, *P01815* unknown protein, *P04220* unknown protein, *PLTP* phospholipid transfer protein, *PRG4* proteoglycan 4, *PSG2* pregnancy specific beta-1-glycoprotein 2 *PSG9* pregnancy-specific beta-1-glyprotein 9, *PSG11* pregnancy-specific beta-1-glyprotein 11, *TAGLN2* Transgelin-2, *THAP4* THAP domain-containing protein 4.

A total of 688 proteins were identified in our proteomics analysis, 25 proteins of them were differentially expressed (p value < 0.05) between cases and controls (Fig. 1). Out of these 25 proteins, 16 were decreased in abundance and 9 were increased in FGR cases. The most highly modulated proteins were: (1) adiponectin (ADIPOQ), an adipocyte-specific protein, which plays a role in protecting against the development of insulin resistance and atherosclerosis^16^, p = 0.003; (2) lymphatic vessel endothelial hyaluronan receptor 1 (LYVE1), an autocrine regulator of cell growth mediated by growth regulators^17^, p = 0.005; (3) Lactotransferrin precursor (LTF), a stimulator of endothelial cell migration and proliferation which has a possible role in the regulation of bone growth^18^, p = 0.010; (4) Galectin-7 (LGALS7), p = 0.010; (5) Phospholipid transfer protein (PLTP), p = 0.013. Some pregnancy specific beta-1-glycoproteins (specifically 2, 9 and 11) were also altered in late-onset FGR mothers, these proteins are mainly secreted by the placenta. In addition, our results uncover the high abundance of many lipoproteins in FGR mothers such as Apolipoprotein C2, Apolipoprotein C3 and Apolipoprotein E as well as fatty acid-binding protein 5. These lipoproteins play a pivotal role in the pathomechanisms of atherosclerosis by the regulation of triglyceride levels^19^. Among the other proteins that are differentially expressed in FGR galectin-3-binding protein, proteoglycan 4 and transgelin-2 which promote cell adhesion^20,21^; epidermal growth factor receptor which may play a role in membrane ruffling and remodeling of the actin cytoskeleton^22^; THAP domain-containing protein 4, platelet glyprotein Ib alpha chain and fibrinogen alpha chain which regulate endothelial cell proliferation and hemostasis^23^; beta-defensin 103 that has an antimicrobial activity; Di-N-acetylchitobiase, involved in the degradation of asparagine-linked glycoproteins and other degradational proteins like ATP synthase subunit g, mitochondrial and Olfactomedin-like-protein 2B. Individual values of the 25 differentially expressed proteins are displayed in Supplementary Table S1.

### Protein–protein interaction network

**Figure 2:**
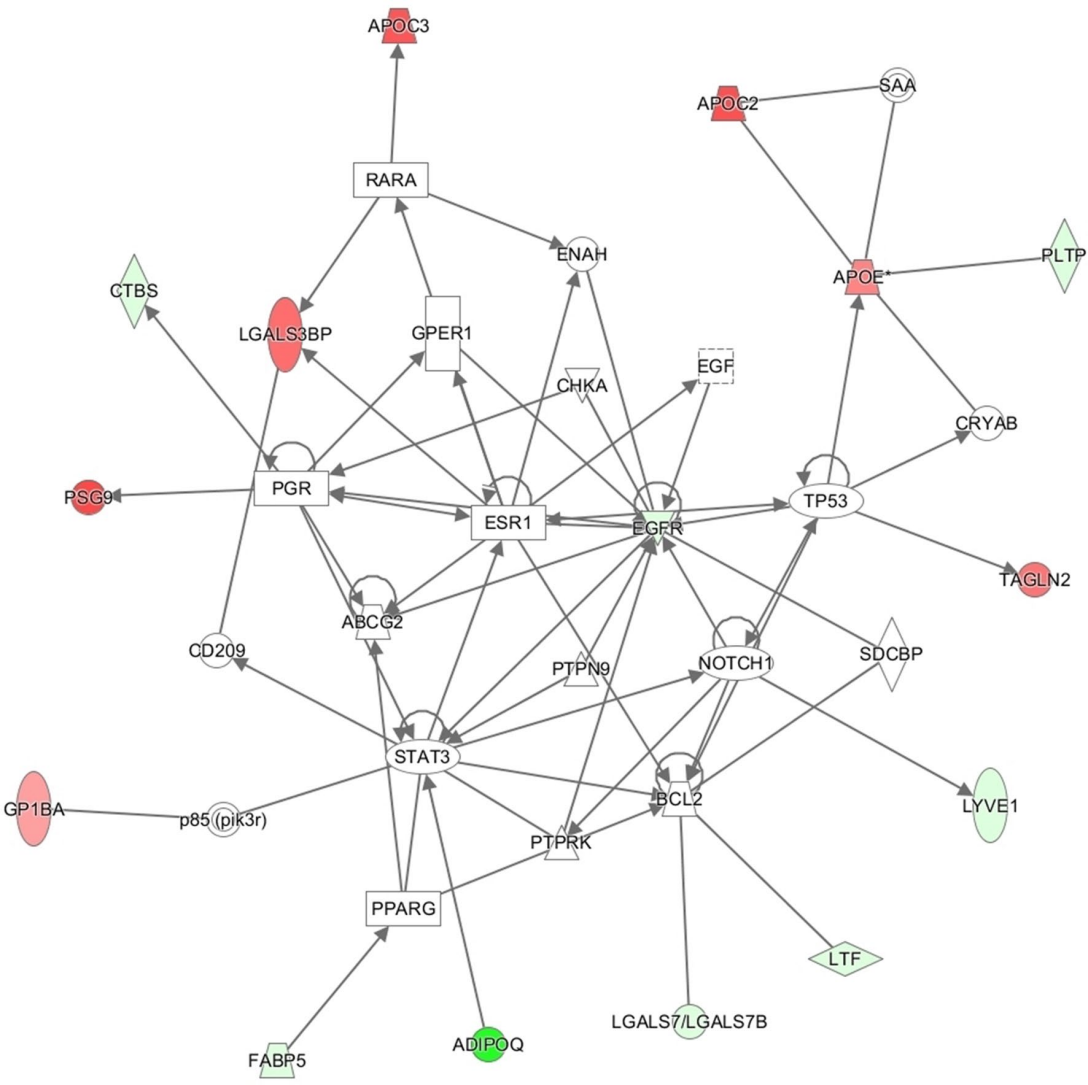
Network analysis combining focused proteins (colored) that correspond to differentially expressed proteins in late-onset fetal growth restriction (green: underexpressed, red: overexpressed) and non-focused proteins that were added by Ingenuity, using knowledge derived data from their own database. *ABCG2* ATP-binding cassette sub-family G member 2, *ADIPOQ* Adiponectin, *APOC2* apolipoprotein C-II, *APOC3* apolipoprotein C-III, *APOE* apolipoprotein E, *BCL2* B-cell lymphoma 2, *CD209* cluster of differentiation 209, *CHKA* choline kinase alpha, *CRYAB* alpha-crystallin B chain, *CTBS* Di-N-acetylchitobiase, *EGF* epidermal growth factor, *EGFR* epidermal growth factor receptor, *ENAH* protein enabled homolog, *ESR1* estrogen receptor 1, *FABP5* fatty acid-binding protein 5, *GP1BA* platelet glyprotein Ib alpha chain, *GPER1* G-protein coupled estrogen receptor 1, *LGALS3BP* galectin-3-binding protein, *LGALS7* galectin-7, *LTF* lactotransferrin, *LYVE1* lymphatic vessel endothelial hyaluronan receptor 1, *NOTCH1* neurogenic locus notch homolog protein 1, *P85 (pirk3r)* phosphatidylinositol 3-kinase, *PGR* progesterone receptor, *PLTP* Phospholipid transfer protein, *PPARG* peroxisome proliferator-activated receptor gamma, *PSG9* pregnancy-specific beta-1-glyprotein 9, *PTPN9* protein tyrosine phosphatase, non-receptor type 9, *PTPRK* protein tyrosine phosphatase receptor type K, *RARA* retinoic acid receptor alpha, *SAA* serum amyloid A, *SDCBP* syndecan binding protein, *STAT3* signal transducer and activator of transcription 3, *TAGLN2* transgelin-2, *TP53* tumor protein p53.

Direct protein–protein interactions among the different components of the network were established by the Ingenuity database (Fig. 2). Neurogenic locus notch homolog protein 1 (NOTCH1) that showed up in this network was highlighted as the most significant putative upstream regulator, meaning that NOTCH1 could be a key regulator of the observed profile. Other proteins such as Signal transducer and activator of transcription 3 (STAT3), Estrogen receptor 1 (ESR1) or ATP-binding cassette sub-family G member 2 (ABCG2) show also many important interactions.

### Biological processes involved

Gene ontology analysis of the corresponding proteins that were statistically different in FGR revealed the involvement of 14 canonical pathways, the top 5 canonical pathways canonical pathways are shown in Table 2. We further studied the potential mechanisms and identified 500 biological processes related to FGR. The most significant biological processes were efflux of cholesterol, efflux of phospholipids, adhesion of blood cells, fatty acid metabolism and dyslipidemia. Most of the top 25 biological processes displayed in Table 3 were related to lipid metabolism or hemostasis.

**Table 2:** Top 5 canonical pathways involved in late-onset fetal growth restriction.

Canonical pathways	p value	Molecules
LXR/RXR Activation	2.17E−07	APOE, APOC2, PLTP, FGA, APOC3
FXR/RXR Activation	2.56E−07	APOE, APOC2, PLTP, FGA, APOC3
LPS/IL-1 Mediated Inhibition of RXR Function	8.29E−05	APOE, APOC2, PLTP, FABP5
Atherosclerosis Signaling	3.66E−04	APOE, APOC2, APOC3
IL-12 Signaling and Production in Macrophages	5.67E−04	APOE, APOC2, APOC3

*APOC2* Apolipoprotein C2, *APOC3* Apolipoprotein C3, *APOE* Apolipoprotein E, *FABP5* Fatty acid-binding protein 5, *FGA* Fibrinogen alpha chain, *FXR* Farsenoid X receptor, *IL-1* Interleukin-1, *IL-12* Interleukin-12, *LPS* Lipopolysaccharide, *LXR* Liver X receptor, *PLTP* Phospholipid transfer protein, *RXR* Retinoid X receptor.

**Table 3:** Top 25 biological processes involved in late-onset fetal growth restriction.

Diseases or Functions Annotation	p value	Molecules
Efflux of cholesterol	4.80E−09	ADIPOQ, APOC2, APOC3, APOE, PLTP
Efflux of phospholipid	4.89E−09	APOC2, APOC3, APOE, PLTP
Adhesion of blood cells	1.96E−07	ADIPOQ, APOE, FGA, GP1BA, LTF, PLTP
Fatty acid metabolism	2.02E−07	ADIPOQ, APOC2, APOC3, APOE, EGFR, LTF, PLTP
Dyslipidemia	1.55E−06	ADIPOQ, APOC2, APOC3, APOE
Adhesion of immune cells	2.49E−06	ADIPOQ, APOE, FGA, LTF, PLTP
Synthesis of fatty acid	2.62E−06	APOC2, APOC3, APOE, EGFR, LTF
Binding of cells	2.77E−06	ADIPOQ, APOE, EGFR, GP1BA, LGALS3BP, LTF
Cell movement of hepatoma cell lines	4.84E−06	ADIPOQ, EGFR, LYVE1, TAGLN2
Concentration of lipid	6.24E−06	ADIPOQ, APOC3, APOE, EGFR, PLTP
Binding of blood cells	1.40E−05	ADIPOQ, APOE, GP1BA, LTF
Adhesion of lymphoma cell lines	1.66E−05	APOE, EGFR, FGA
Homeostasis of lipid	1,90E−05	APOC2, APOC3, APOE
Adhesion of tumor cell lines	3.28E−05	APOE, EGFR, FGA, GP1BA, LTF
Progression of digestive organ tumor	3.47E−05	APOE, EGFR
Progression of carcinoma	4.46E−05	APOE, EGFR
Binding of myeloid cells	4.68E−05	ADIPOQ, APOE, LTF
Aggregation of cells	4.83E−05	EGFR, FGA, GP1BA, PSG2
Hyperlipidemia	5.39E−05	APOC2, APOC3, APOE
Binding of macrophages	6.80E−05	ADIPOQ, APOE
Fibrinolysis	8.16E−05	FGA, GP1BA
Lower respiratory tract disorder	9.59E−05	EGFR, FABP5, LTF, PLTP
Fibrin clot	9.64E−05	FGA, GP1BA
Homeostasis of triacylglycerol	9.64E−05	APOC2, APOC3
Binding of fibroblasts	1.12E−04	APOE, LGALS3BP

*ADIPOQ* Adiponectin, *APOC2* Apolipoprotein C-II, *APOC3* Apolipoprotein C-III, *APOE* Apolipoprotein E, *EGFR* Epidermal growth factor receptor, *FABP5* Fatty acid-binding protein 5, *FGA* Fibrinogen alpha chain, *GP1BA* Platelet glyprotein Ib alpha chain, *LGALS3BP* Galectin-3-binding protein, *LTF* Lactotransferrin precursor, *LYVE1* Lymphatic vessel endothelial hyaluronan receptor 1, *PLTP* Phospholipid transfer protein, *PSG2* Pregnancy specific beta-1-glycoprotein 2.

## Discussion

This is the first study that focuses on maternal proteomic profile in pregnancies complicated by the late-onset form of FGR. The results of the present study elucidate that lipid metabolism is disturbed in mothers from pregnancies complicated by late-onset FGR compared to healthy pregnancies. Furthermore, our results indicate that NOTCH1 could be an important regulator of the observed profile.

The findings of our study demonstrate that late-onset FGR has a proteomic signature in maternal plasma similarly to the previous observations that focused on the early-onset form of this disorder^15^ or mixed up early and late-onset cases^13,14^. A vast majority of the identified proteins and biological processes are related to lipid metabolism which is in line with prior studies^10,24,25^. Among the differentially expressed proteins in late-onset FGR, the highest magnitude of change was observed in Adiponectin that was underexpressed in FGR mothers. This observation might reflect a failure in the physiological response to pregnancy demands since Adiponectin concentrations are usually elevated in healthy pregnancies due to pregnancy related “Adiponectin resistance”^26^.

In light of the protein–protein interactions observed in late-onset FGR, we can hypothesize that different biological processes related to lipid metabolism and homeostasis have an impact on other regulator proteins. These proteins may play an essential role in the pathogenesis of late-onset FGR due to their close relationship with the development of the extravillous trophoblast lineage in the human placenta (NOTCH1)^27^, the regulation of trophoblast invasion and the expression and activity of placental amino acid transporters (STAT3)^28^ or the differentiation of estrogen-dependent cells (ESR)^29^. Indeed, previous studies have demonstrated alterations in the immunoreactivity and localization of NOTCH proteins as well as decreased STAT3 in placentas from pregnancies complicated by FGR suggesting a contribution of these disruptions in trophoblast differentiation and function^30,31^. In the present study, NOTCH1 was the most featured regulator in the spotted network linking the disturbed profile of lipid metabolism with placental growth and being a potential target for future therapeutic agents.

Furthermore, our gene ontology analysis has revealed that the top canonical pathways and biological processes involved in late-onset FGR are mostly related to the efflux of cholesterol and phospholipids. Indeed, multiple pathways share similar identified molecules where the lipoproteins Apolipoprotein C2, Apolipoprotein C3 and Apolipoprotein E are central. Thus, co-activation of parallel pathways seems to have occurred in FGR mothers. The most significant pathway was LXR/RXR activation, a fundamental pathway in the balance of cholesterol levels^18^ and known to have a protective function against dysregulated fetoplacental lipid homeostasis^32^. In addition, FXR/RXR activation and LPS/IL-1 Mediated Inhibition of RXR Function were also initiated, which might affect several functions since FXR is a metabolic regulator and cell protector against oxidative stress^33^. The same lipoproteins are involved in atherosclerosis and IL-12 signaling replicating the link between inflammation, lipid dysregulation and endothelial cell dysfunction. In fact, oxidative stress, inflammation and placental thrombosis are likely to interrupt the placental ability to transfer the necessary nutrients and oxygen to the fetus and therefore impede the normal fetal growth^34–37^.

The current study focused on the late-onset form of this disorder revealing specific pathways and key player proteins that can provide further insights into the pathophysiology of late-onset FGR. In fact, lipid metabolism during the normal pregnancy is essential to provide the necessary fatty acids for fetal growth^38^. It is widely accepted that placental insufficiency is the main culprit in FGR resulting from shallow trophoblast invasion during the early stages of gestation^7^. Indeed, many placental enzymes involved in the supply of fatty acids to the growing fetus, like endothelial lipase and lipoprotein lipase, have been described to be dysregulated in FGR pregnancies^39^. Thus, it seems plausible that maternal poor response to pregnancy demands of lipids and fatty acids may contribute to the placental dysfunction and suboptimal fetal growth. In addition, we observed a favored proinflammatory status in the studied cases of late-onset FGR supporting the existence of an inflammatory bias in this disorder^40^.

This study has some strengths and limitations that merit a comment. All the pregnancies included in this study were recruited prospectively, well selected and characterized to constitute homogenous groups of late-onset FGR cases and controls. Cases and controls were matched by gestational age at maternal blood sampling. Moreover, this was a comprehensive proteomics study not only revealing the different proteins in late-onset FGR but also the protein–protein interactions and the involved biological processes. The center of our analysis was to identify the pathophysiological pathways that may play a role in this form of FGR, thus we opted for a wider look at the results without applying a statistical correction for multiple comparisons. On the other hand, we acknowledge the small sample size of our study and the importance of future validation of our findings in larger cohorts.

In conclusion, the present study indicates that lipid metabolism dysregulation plays a vital role in pregnancies complicated by late-onset FGR. Importantly, our findings highlight the central regulator of the observed profile being NOTCH1. These results enhance our understanding of the pathophysiology of late-onset FGR which remains poorly defined. Furthermore, they may constitute a starting point for future studies to investigate the potential therapeutic targets of the involved pathways.

## Methods

### Study design

We conducted a prospective case–control study in the Departments of Maternal–Fetal Medicine at BCNatal (Barcelona, Spain) between July and October 2016. The study population included 5 singleton pregnancies diagnosed with late-onset FGR which was defined as an estimated fetal weight and birthweight below the 10th centile^2^. Late-onset refers to delivery occurring after 37 weeks of gestation. Uncomplicated pregnancies with appropriate fetal growth for gestational age-defined as estimated fetal weight and birthweight above the 10th centile were randomly selected from our general population to be included as controls and frequency paired with cases by gestational age at maternal blood sampling (± 2 weeks). In all pregnancies, gestational age was calculated based on crown–rump length measurement on first-trimester ultrasound^41^ and weight centiles were assigned according to local standards^42^. Pregnancies with congenital malformations, chromosomal abnormalities or intrauterine infection were excluded. The study was conducted in accordance with the principles of the Helsinki declaration. The study protocol has been approved by the local ethics committee (Comité Ético de Investigación Clinica, Hospital Clinic, Barcelona) number HCB/2016/0253. Participating patients provided their written informed consent.

### Data collection and study protocol

The following data were recorded upon enrollment: maternal age, ethnicity, known chronic disease (i.e. hypertension, diabetes mellitus), parity, obstetric history, mode of conception and smoking status. Feto-placental Doppler parameters were obtained in the last 2 weeks of pregnancy, including the uterine arteries^43^, the umbilical artery^44^, and the fetal middle cerebral artery pulsatility indices^44^, with the calculation of the cerebroplacental ratio^45^. These values were normalized into z scores accordingly and considered abnormal if > 95th centile for uterine arteries mean and umbilical artery pulsatility indices and < 5th centile for the middle cerebral artery pulsatiliy index and the cerebroplacental ratio^43–45^. At the time of delivery, gestational age, birthweight, birthweight centile, Apgar scores, umbilical artery pH and perinatal mortality were recorded. In addition, maternal blood samples were collected for subsequent proteomic analysis.

### Maternal blood sampling

Maternal blood samples were drawn from peripheral veins within 2 h after delivery and collected in EDTA-treated tubes. Plasma was separated by centrifugation at 1500*g* for 10 min at 4 °C, and samples were immediately stored at − 80 °C until analyzed.

### Proteomics technique

Before proteomic analysis, the depletion of fourteen highly and medium abundant proteins was performed using Seppro IgY14 and Seppro SuperMix columns following the manufacturer’s instructions. Afterwards, samples were processed for tandem mass tag (TMT) before acquisition on a nanoscale liquid chromatography coupled to tandem mass spectrometry (2D nano LC–MS/MS) analysis from Thermo Fisher. Protein identification/quantification was performed on Proteome Discoverer software v.1.4.0.288 (Thermo Fisher) by Multidimensional Protein Identification Technology. On initial proteomic analysis, readers were blinded to each patient’s status. Detailed methodology is provided as supplementary information.

### Statistical analysis

Clinical characteristics of the study population were summarized as median (interquartile range) or percentages for continuous and categorical variables respectively. The analysis was performed using STATA 14.2 (StataCorp LLC, Texas, USA) including the use of Mann Whitney U test and Fisher exact test for continuous and categorical variables respectively. All reported p values are two-sided. Differences were considered significant when p < 0.05.

For proteomics data, differentially expressed proteins were determined using R package “limma”^46^. Data were preprocessed, normalized and a moderated t-test was applied (p < 0.05). Network analysis was generated through the use of QIAGEN’s Ingenuity Pathway Analysis (IPA), QIAGEN Inc., (https://www.qiagenbioinformatics.com/products/ingenuity-pathway-analysis). Networks combined focused proteins that correspond to differentially expressed proteins which were detected by “limma” analysis and non-focused proteins that were added by IPA, using knowledge derived data from their own database. To gain a further insight into the potential mechanisms involved, the identified proteins were mapped to IPA database.

## Supplementary information

Supplementary Information S1.

Supplementary Information S2.

## Data Availability

The proteomics quantification data reported in this study are available as supplementary information.

## References

[1] Lee ACC (2013). National and regional estimates of term and preterm babies born small for gestational age in 138 low-income and middle-income countries in 2010. Lancet Glob. Health.

[2] Figueras F, Gratacós E (2014). Update on the diagnosis and classification of fetal growth restriction and proposal of a stage-based management protocol. Fetal Diagn. Ther..

[3] Barker DJ, Osmond C, Golding J, Kuh D, Wadsworth ME (1989). Growth in utero, blood pressure in childhood and adult life, and mortality from cardiovascular disease. BMJ.

[4] Crispi F, Miranda J, Gratacós E (2018). Long-term cardiovascular consequences of fetal growth restriction: Biology, clinical implications, and opportunities for prevention of adult disease. Am. J. Obstet. Gynecol..

[5] Løhaugen GCC (2013). Small for gestational age and intrauterine growth restriction decreases cognitive function in young adults. J. Pediatr..

[6] Savchev S (2014). Evaluation of an optimal gestational age cut-off for the definition of early-and late-onset fetal growth restriction. Fetal Diagn. Ther..

[7] Figueras F (2018). Diagnosis and surveillance of late-onset fetal growth restriction. Am. J. Obstet. Gynecol..

[8] Gardosi J, Madurasinghe V, Williams M, Malik A, Francis A (2013). Maternal and fetal risk factors for stillbirth: Population based study. BMJ.

[9] Burton GJ, Jauniaux E (2018). Pathophysiology of placental-derived fetal growth restriction. Am. J. Obstet. Gynecol..

[10] Miranda J (2018). Metabolic profiling and targeted lipidomics reveals a disturbed lipid profile in mothers and fetuses with intrauterine growth restriction. Sci. Rep..

[11] Phizicky E, Bastiaens PIH, Zhu H, Snyder M, Fields S (2003). Protein analysis on a proteomic scale. Nature.

[12] Horgan RP, Clancy OH, Myers JE, Baker PN (2009). An overview of proteomic and metabolomic technologies and their application to pregnancy research. BJOG Int. J. Obstet. Gynaecol..

[13] Gupta MB (2006). Altered proteome profiles in maternal plasma in pregnancies with fetal growth restriction: Haptoglobin α2 isoform as a potential biomarker. Clin. Proteom..

[14] Auer J (2010). Serum profile in preeclampsia and intra-uterine growth restriction revealed by iTRAQ technology. J. Proteom..

[15] Wölter M (2016). Proteoform profiling of peripheral blood serum proteins from pregnant women provides a molecular IUGR signature. J. Proteom..

[16] Lihn AS, Pedersen SB, Richelsen B (2005). Adiponectin: Action, regulation and association to insulin sensitivity. Obes. Rev..

[17] Huang SS (2003). Cloning, expression, characterization, and role in autocrine cell growth of cell surface retention sequence binding protein-1. J. Biol. Chem..

[18] Murthy S, Born E, Mathur SN, Field FJ (2002). LXR/RXR activation enhances basolateral efflux of cholesterol in CaCo-2 cells. J. Lipid Res..

[19] Hořejší B, Češka R (2000). Apolipoproteins and atherosclerosis. Apolipoprotein E and apolipoprotein(a) as candidate genes of premature development of atheroselerosis. Physiol. Res..

[20] Adhesion C, Inohara H, Akahani S, Koths K, Raz A (1996). Interactions between Galectin-3 and Mac-2-binding. Cancer Res..

[21] Elsafadi M (2016). Transgelin is a TGFβ-inducible gene that regulates osteoblastic and adipogenic differentiation of human skeletal stem cells through actin cytoskeleston organization. Cell Death Dis..

[22] Wieduwilt MJ, Moasser MM (2008). The epidermal growth factor receptor family: Biology driving targeted therapeutics. Cell. Mol. Life Sci..

[23] Lopez JA (1988). The α and β chains of human platelet glycoprotein Ib are both transmembrane proteins containing a leucine-rich amino acid sequence. Proc. Natl. Acad. Sci. USA.

[24] Wadsack C (2007). Intrauterine growth restriction is associated with alterations in placental lipoprotein receptors and maternal lipoprotein composition. Am. J. Physiol. Endocrinol. Metab..

[25] Pecks U (2012). The evaluation of the oxidative state of low-density lipoproteins in intrauterine growth restriction and preeclampsia. Hypertens. Pregnancy.

[26] Mazaki-Tovi S (2007). Maternal serum adiponectin levels during human pregnancy. J. Perinatol..

[27] Haider S (2016). Notch1 controls development of the extravillous trophoblast lineage in the human placenta. Proc. Natl. Acad. Sci. USA.

[28] Zhang Z, Wang X, Wang J, Zhang L (2018). The decreased expression of Stat3 and p-Stat3 in preeclampsia-like rat placenta. J. Mol. Histol..

[29] Bukovsky A (2003). Placental expression of estrogen receptor beta and its hormone binding variant—comparison with estrogen receptor alpha and a role for estrogen receptors in asymmetric division and differentiation of estrogen-dependent cells. Reprod. Biol. Endocrinol..

[30] Sahin Z, Acar N, Ozbey O, Ustunel I, Demir R (2011). Distribution of Notch family proteins in intrauterine growth restriction and hypertension complicated human term placentas. Acta Histochem..

[31] Borg AJ (2015). Decreased STAT3 in human idiopathic fetal growth restriction contributes to trophoblast dysfunction. Reproduction.

[32] Nikolova V (2017). Changes in LXR signaling influence early-pregnancy lipogenesis and protect against dysregulated fetoplacental lipid homeostasis. Am. J. Physiol. Endocrinol. Metab..

[33] Wang YD, Chen WD, Moore DD, Huang W (2008). FXR: A metabolic regulator and cell protector. Cell Res..

[34] Al-Azemi M, Raghupathy R, Azizieh F (2017). Pro-inflammatory and anti-inflammatory cytokine profiles in fetal growth restriction. Clin. Exp. Obstet. Gynecol..

[35] Paules C (2019). Distinctive patterns of placental lesions in preeclampsia versus fetal growth restriction and their association with fetoplacental Doppler. Ultrasound Obstet. Gynecol..

[36] Biri A (2007). Role of oxidative stress in intrauterine growth restriction. Gynecol. Obstet. Invest..

[37] Youssef L (2020). Hemopexin and α1-microglobulin heme scavengers with differential involvement in preeclampsia and fetal growth restriction. PLoS One.

[38] Herrera E (2002). Lipid metabolism in pregnancy and its consequences in the fetus and newborn. Endocrine.

[39] Gauster M (2007). Dysregulation of placental endothelial lipase and lipoprotein lipase in intrauterine growth-restricted pregnancies. J. Clin. Endocrinol. Metab..

[40] Raghupathy R, Al-Azemi M, Azizieh F (2012). Intrauterine growth restriction: Cytokine profiles of trophoblast antigen-stimulated maternal lymphocytes. Clin. Dev. Immunol..

[41] Robinson H, Fleming J (1975). A critical evaluation of sonar crown-rump length measurements. Br. J. Obstet. Gynaecol..

[42] Figueras F (2008). Customized birthweight standards for a Spanish population. Eur. J. Obstet. Gynecol. Reprod. Biol..

[43] Gómez O (2008). Reference ranges for uterine artery mean pulsatility index at 11–41 weeks of gestation. Ultrasound Obstet. Gynecol..

[44] Arduini D, Rizzo G (1990). Normal values of pulsatility index from fetal vessels: A cross-sectional study on 1556 healthy fetuses. J. Perinat. Med..

[45] Baschat AA, Gembruch U (2003). The cerebroplacental Doppler ratio revisited. Ultrasound Obstet. Gynecol..

[46] Ritchie ME (2015). Limma powers differential expression analyses for RNA-sequencing and microarray studies. Nucleic Acids Res..

