# Supplementary material for: Maternal proteomic profiling reveals alterations in lipid metabolism in late-onset fetal growth restriction
Source: Sci Rep. 2020 Dec 3;10:21033. doi: 10.1038/s41598-020-78207-3 (PMC7713381; doi:10.1038/s41598-020-78207-3)
Supplement: Supplementary file 2 — Supplementary Information S2. [file 41598_2020_78207_MOESM2_ESM.pdf]

Maternal proteomic profiling reveals alterations in lipid metabolism in late-onset fetal growth restriction

Cristina Paules[1,2#], Lina Youssef[1#], Jezid Miranda[1], Francesca Crovetto[1], Josep Maria Estanyol[3], Guerau Fernandez[4], Fatima Crispi[1,5]*, Eduard Gratacós[1,5]

[1]BCNatal | Fetal Medicine Research Center (Hospital Clínic and Hospital Sant Joan de Déu), Institut d'Investigacions Biomèdiques August Pi i Sunyer (IDIBAPS), University of Barcelona, Barcelona, Spain.

[2]Instituto de Investigación Sanitaria Aragón (IISAragon), Zaragoza, Spain

[3]Centres Cientifics i Tecnològics (CCiTUB), University of Barcelona, Barcelona, Spain.

[4]Bioinformatics Unit, Genetics and Molecular Medicine Service, Hospital Sant Joan de Déu, Esplugues de Llobregat, Spain.

[5]Centre for Biomedical Research on Rare Diseases (CIBER-ER), Madrid, Spain.

#Both authors have contributed equally to this work.

*Corresponding author: Fatima Crispi, MD, PhD, Department of Maternal-Fetal Medicine (ICGON), Hospital Clínic, Sabino de Arana 1, 08028 Barcelona, Spain; phone: +34 93 227 9333; fax: +34 93 227 5612; e-mail: fcrispi@clinic.cat.

Supplementary Information

Proteomics technique

Sample preparation

<u>Columns IgY14 seppro</u>

This column based on antibody (YgY)-antigen interactions is specifically designed to remove fourteen highly abundant proteins from human fluids such as serum or plasma.

We filtered samples 1mg from each and per duplicate. After aconditionate the column, sample duplicates were loaded in order to wash and elute proteins following manufacturer protocol Seppro IgY14 Spin columns catalog number SEP010.

Flow through, FT, was kept in order to be loaded onto the Seppro SuperMix LC2 Column,(40ul of this FT are separated). Then we wash the column 4 times .The first wash was mixed with the FT (low abundant proteins), these will go to the Columns Seppro SuperMix LC2 Column. We discard the rest of the washes (proteins bounded non-specifically bound), and finally we get in the elution step after washing 4 times with the corresponding buffer the most abundant proteins.

<u>Seppro SuperMix LC2 Column</u>

We load the FT and first wash onto this column following manufacturer protocol Seppro SuperMix LC2 column catalog number SEP050. Working at a flow rate of 1.5ml/min. we collected for 15 minutes the flow through and first wash, we discarded the following 9 minutes gradient, and we collected and kept the Elution part of the last 7 minutes.

Then in order to minimize the final volume of aprox 13ml we use a centricon tube to obtain approximately 1.5ml followed by the precipitation with TCA 10%, the FT and Elution of the supermix, and the previous 40ul from Yg14 elution.

Digestion

These pellets are reconstituted in 20 ul (TrisHCL 100mM TEAB, 0,1%SDS, 10mM DTT) and after reduction they were alkylated with IAA .After reaction we precipitated proteins using 6 volumes of ice cold acetone. The resulting pellet was reconstitute in 10ul and digested with 3ug of trypsin. Samples were dried afterwards.

Labeling sixplex

Dried samples were reconstituted in 50ul 100mM of TEAB, 8.3 ul from each were taken in order to make the pool of samples. Samples were labeled after adding 41 ul of acetonitrile to the tags and taking only 20.5ul for labeling as follows:

**RUN 1**		**RUN 2**	
**SAMPLE**	**TAG**	**SAMPLE**	**TAG**
7	127N	62	127N
9	128N	70	128N
10	129N	72	129N
33	130N	74	130N
40	131	77	131

Tag 126 ALL SAMPLES CONTROL (pool of equal amount of each sample)

After labeling samples were fractionated on a SCX column, fractions were collected under gravity following these steps:

**SCX COLUMN**	**STEPS**	**VOLUM ul**	**PROTOCOLO-SCX-OASIS**
	Activation	1000	ACN-100%
	Equilibration	1000	5mM(F-NH4-Ph3_25%ACN)
		1000	5mM(F-NH4-Ph3_25%ACN)
	Load	1000	Muestra
	Fraction	1000	5mM(F-NH4-Ph3_25%ACN)
	Fraction	600	200mM(F-NH4-Ph3_25%ACN)
	Fraction	600	350mM(F-NH4-Ph3_25%ACN)
	Fraction	600	500mM(F-NH4-Ph3_25%ACN)
	Fraction	600	1000mM(F-NH4-Ph3_25%ACN)
	Fraction	600	1500mM(F-NH4-Ph3_25%ACN)
	Fraction	600	500mM(F-NH4-Ph3_25%ACN_1MKCL)
	Fraction	600	500mM(F-NH4-Ph3_25%ACN_1.5MKCL)

	Fraction	600	H_2O
	Fraction	600	100%ACN

Each fraction was cleaned up using c18 sping columns. The eluate was analyze afterwards by:

Analysis by reverse phase chromatography- MS/MS:

<u>Chromatography</u>

Reversed phase nano LC MS/MS setup comprised of(nanoUltra 2DEksigent) coupled to an LTQ Velos-Orbitrap mass spectrometer (Thermo scientific, San Jose, CA). Peptides were loaded onto a trap column C18 , 8ul, (L 2cm, 100um ID, 5um,120 Å,thermo scientific). Gradient was applied on line with an analytical column (L 15cm, 75um ID, 3um, 100 Å, Thermoscientific). Buffer System, buffer A (97% H_2O-3%ACN, 0.1%Formic acid) and buffer B (97% ACN-3%H_2O, 0.1%Formic acid). For the peptide mixtures the following gradient was applied,(0–4 min 0% to 5% B, 4–150 min 5% to 35% B, 150-155min 35% to100% B, 155-170 min 100% to 100% B at a flow rate of 400nl/min.

<u>Mass spectrometry:</u>

Peptides were loaded directly onto a nano ESI spray source and were sprayed directly into a hybrid linear ion trap - Orbitrap mass spectrometer (LTQ-Orbitrap Velos, Thermo Fisher Scientific). Peptides were analyzed using a “high/high” acquisition strategy, detecting at high resolution in the Orbitrap and analyzing the subsequent fragments from HCD also in the Orbitrap. Survey scan (MS) spectra were recorded in the Orbitrap at 30.000 resolution at m/z of 400. The 30 most intense signals in the survey scan for each acquisition cycle were isolated with an m/z window of 2. 1+ ions were excluded from fragmentation. Fragmentation (MS2) spectra were acquired in the Orbitrap at 7.500

resolution at m/z of 400. Dynamic exclusion was enabled with 90 s exclusion time and repeat count equal to 1

Search Analysis

All raw data from each fraction of each sample were combined. Raw data were searched against the human subset of UniProt home made database (HUMAN_Tryp_UP_SP_R_2016_03.fasta; 20155 total proteins in the database) using Proteome Discoverer 1.4(version 1.4) using search parameters: MS accuracy, 20 ppm; MS/MS accuracy, 0.06 Da; enzyme, trypsin; specificity, fully tryptic; allowed number of missed cleavages, 4; fixed modifications, Carbamidomethyl / +57.021 Da (C) ; variable modifications, N-Terminal Modification TMT6Plex / +229.163 Da (Any N-Terminus) ,Oxidation / +15.995 Da (M) and TMT6Plex / +229.163 Da (K). Only peptides with 0.05 FDR were taken for further analyses.

Results

A total of 688 proteins were identified in our proteomics analysis, 25 proteins of them were differentially expressed (p-value <0.05) between cases and controls. Individual values of the 25 differentially expressed proteins are displayed in Supplementary Table 1.

Supplementaru Table 1: Individual values of differentially expressed proteins in late-onset fetal growth restriction.

ADIPOQ, Adiponectin; APOC2, Apolipoprotein C-II; APOC3, Apolipoprotein C-III; APOE, Apolipoprotein E; ATP5L, ATP synthase subunit g mitochondrial; CTBS, Di-N-acetylchitobiase; DEFB103A, Beta-defensin 103; EGFR, Epidermal growth factor receptor; FABP5, Fatty acid-binding protein 5; FGA, Fibrinogen alpha chain; GP1BA, Platelet glyprotein Ib alpha chain; LGALS3BP, Galectin-3-binding protein; LGALS7, Galectin-7; LTF, Lactotransferrin precursor; LYVE1, Lymphatic vessel endothelial hyaluronan receptor 1; OLFML2B, Olfactomedin-like-protein 2B; P01815, unknown protein; P04220, unknown protein; PLTP, Phospholipid transfer protein; PRG4, Proteoglycan 4; PSG2, Pregnancy specific beta-1-glycoprotein 2; PSG9, Pregnancy-specific beta-1-glyprotein 9; PSG11, Pregnancy-specific beta-1-glyprotein 11; TAGLN2, Transgelin-2; THAP4, THAP domain-containing protein 4.

Uniprot	Gene	Control 1	Control 2	Control 3	Control 4	Control 5	Case 1	Case 2	Case 3	Case 4	Case 5
Q15848	ADIPOQ	0.7790	1.1414	0.2733	0.6702	0.0834	0.4457	0.7301	1.2878	0.5159	0.2026
Q9Y5Y7	LYVE1	0.9450	0.8909	0.4862	1.2315	0.1318	0.8197	0.8905	0.7912	0.5673	0.2908
P02788	LTF	1.2977	1.1181	0.5254	1.0991	0.0877	0.8368	0.8600	0.8681	0.7341	0.2163
P47929	LGALS7	2.3078	0.8974	0.1996	1.0144	0.4352	0.7129	0.7286	0.6413	0.9582	0.3721
P55058	PLTP	1.0936	1.1562	0.4810	0.7128	0.1015	0.7321	1.3599	0.9341	0.6239	0.1519
P11465	PSG2	1.4346	0.5130	0.9009	1.2320	0.0624	0.8570	1.1380	0.8019	0.5647	0.1019
P01815		2.571	3.6955				0.4798	1.543	3.018		
Q08380	LGALS3BP	0.6241	0.4792	0.5040	1.9486	0.0875	0.9348	1.3216	0.5906	0.6474	0.1316

Q9UQ72	PSG11	0.7549	0.6477	0.4524	1.2356	0.1029	0.7658	0.8241	0.7105	0.8050	0.2309
Q92954	PRG4	1.1187	1.1565	0.3873	0.7295	0.0752	0.9792	1.0263	0.9853	0.5723	0.1248
P37802	TAGLN2	0.7218	0.8297	0.4040	0.8645	0.1029	0.7169	1.2887	0.9393	0.9104	0.1123
Q00887	PSG9	0.7447	0.6079	0.7543	1.6964	0.0938	1.3187	0.7398	0.6747	0.6149	0.1401
P00533	EGFR	2.5192	3.2601	0.2576	1.0566	0.3178	0.4569	1.6073	3.8273	0.5212	0.1294
P02655	APOC2	0.6384	0.6224	0.6511	0.7617	0.0737	1.2346	1.3229	1.0289	0.7275	0.0794
Q8WY91	THAP4	0.5256	0.7599	0.6919	0.6885	0.0639	2.1474	1.3721	1.0886	0.8033	0.1248
Q01469	FABP5	5.9443	3.3904	0.2112	1.0438	0.3019	0.6989	1.3527	3.8485	0.5838	0.1805
P81534	DEFB103A	1.3705	1.2728	0.1890	0.8367	0.0964	0.7579	0.7008	0.8286	0.4172	0.0955
Q01459	CTBS	1.0303	0.8417	0.6043	1.0696	0.1478	1.1117	1.1023	0.7864	0.6938	0.2255
O75964	ATP5L	2.9878	3.8323				0.6890	1.3024	3.9604		
Q68BL8	OLFML2B	2.7371	2.2255	0.2416	0.8291	0.2166	0.4077	1.1486	4.096	0.5400	0.1594
P02656	APOC3	0.4129	0.4656	0.5708	0.7394	0.0456	0.7144	1.6174	1.2422	0.5410	0.0802
P04220		1.6210	0.8141	0.4910	0.4952	0.1444	0.7494	1.0666	0.8620	0.7509	0.1050
P07359	GP1BA	0.8975	0.5194	0.6430	1.4352	0.1274	0.9126	1.7756	0.6123	0.9544	0.2212

P02671	FGA	1.1661	0.9503	0.1857	0.3812	0.0381	0.7657	0.4651	1.4542	0.2524	0.0509
P02649	APOE	0.7395	0.9818	0.7863	0.7704	0.0827	1.5375	1.6703	0.8461	0.7320	0.1040
